# Supplementary material for: Association between oral microbiome and seven types of cancers in East Asian population: a two-sample Mendelian randomization analysis
Source: Front Mol Biosci. 2023 Nov 21;10:1327893. doi: 10.3389/fmolb.2023.1327893 (PMC10702768; doi:10.3389/fmolb.2023.1327893)
Supplement: Supplementary file 1 [file DataSheet1.ZIP › Supplementary Materials/MR plots for tongue/tongue═╝/Lung cancer/pheno.2721_to_lung cancer_forest.pdf]

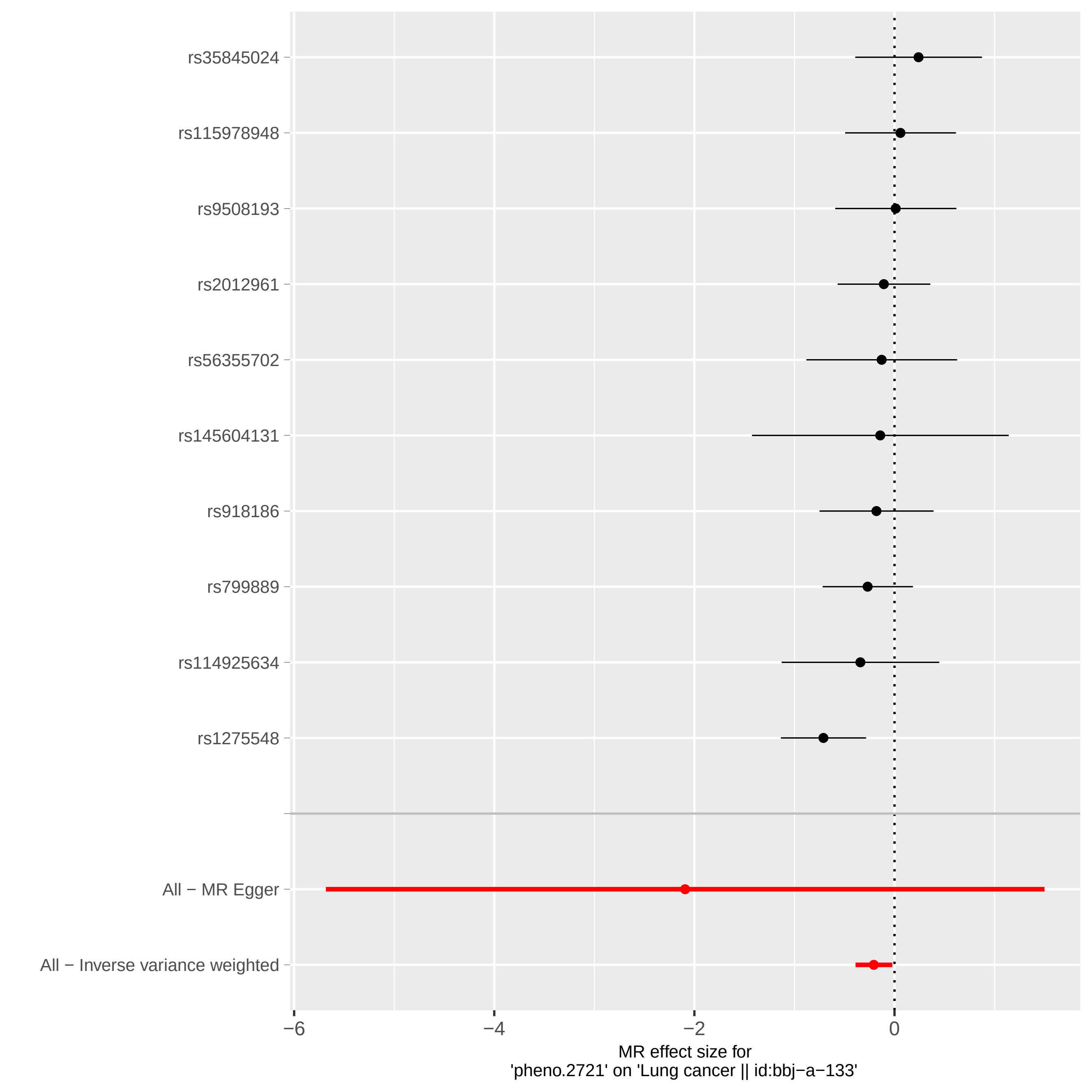

rs35845024
rs115978948
rs9508193
rs2012961
rs56355702
rs145604131
rs918186
rs799889
rs114925634
rs1275548
All − MR Egger
All − Inverse variance weighted
−6
−4
−2
0
MR effect size for
'pheno.2721' on 'Lung cancer || id:bbj−a−133'
